# Personal

**Published:** 1904-04

**Authors:** 


### PERSONAL.

HON. T. GUILFORD SMITH, of Buffalo, who was a life member of the Board of Regents of the University of the State of New York as formerly constituted, has been elected one of the eleven regents who are to govern the educational system under the new plan. Regent Smith, in the drawing for terms of service, was chosen for eight years.

---

DR. A. VANDER VEER, of Albany, a member of the former board of regents, has been elected to the new board and drew to serve one year. The interests of the medical profession will not be neglected while Regent Vander Veer continues a member of the board. Dr. Vander Veer has recently been appointed surgeon-in-chief of the Albany hospital. He desired to retire after so many years' service as attending surgeon, but the hospital governors prevailed upon him to accept this new office which was created for him.

---

DR. LEONARD WOOD, brigadier-general United States army, was recently confirmed by the senate as major general, after a great flourish of opposition lasting some months, the vote standing 45 to 16. General Wood is at present serving in the Philippines, and has well earned this promotion. He has probably been instrumental in saving more human lives than any other major general in the army.

---

Dr. Harvey R. Gaylord, of Buffalo, who recently spent some months in Europe, has returned and removed to No. 478 Delaware avenue.

---

Dr. Andrew S. Draper, president of the University of Illinois, has been elected by the legislature commissioner of education of the state of New York under the new law. Dr. Draper was formerly superintendent of education and is familiar with the schools and colleges in this state. He assumed the duties of his new office April 1, 1904.

---

Dr. Roswell Park, of Buffalo, sailed for Europe on the Blucher, March 24, and will attend the German Surgical Congress at Berlin, April 4-9, 1904. He expects to be absent about six weeks.

---

Dr. James Wright Putnam, of Buffalo, delivered an address, March 17, 1904, at the Twentieth Century Club, to the students of Buffalo Seminary, taking for his subject, Rest, an essential to right and joyous living.

---

Dr. Reger Cutting, a graduate of the University of Buffalo, 1902, and who afterward served as house surgeon at the Emergency Hospital, has located at Attica, N. Y., for the practice of his profession.

---

Dr. David Wheeler, of Buffalo, has been appointed a member of the staff of the Buffalo Hospital of the Sisters of Charity, vice Dr. Byron H. Daggett, deceased.

---

Dr. Frederick Petersen, of New York, president of the State Lunacy Commission, has tendered his resignation to the Governor. Dr. Petersen states a cogent reason for his action—namely, that his practice will not permit him to remain longer in a place that demands so much time. When will the commonwealth learn that in order to obtain and retain the services of competent medical officers it must offer adequate remuneration?

